# Liraglutide protects pancreatic β-cells against free fatty acids *in vitro* and affects glucolipid metabolism in apolipoprotein E^−/−^ mice by activating autophagy

**DOI:** 10.3892/mmr.2015.3944

**Published:** 2015-06-16

**Authors:** JIA WANG, JIE WU, HONG WU, XINGZHEN LIU, YINGJIAN CHEN, JIANYING WU, CHENGJIN HU, DAJIN ZOU

**Affiliations:** 1 Department of Endocrinology, Changhai Hospital, The Second Military Medical University, Shanghai 200433, P.R. China; 2 Department of Internal Medicine, Hangzhou Sanatorium of Nanjing Military Region, Hangzhou, Zhejiang 310000, P.R. China; 3 Department of Laboratory Diagnostics, General Hospital of Jinan Military District, Jinan, Shandong 250031, P.R. China

**Keywords:** liraglutide, pancreatic β-cells, glucolipid metabolism, protective effect

## Abstract

The aim of the present study was to determine whether liraglutide (LRG), a long acting glucagon-like peptide 1 analogue, exerted a protective effect on free fatty acid (FFA)-treated pancreatic β-cells via activating autophagy. INS-1 insulinoma pancreatic islet cell lines were treated with FFA and the levels of cell necrosis, apoptosis and autophagy were detected using an MTT assay, flow cytometry and electron microscopy (ECM). A type 2 diabetes mellitus mouse model was established through treatment of mice with a high-fat diet for 8 weeks and injection of streptozotocin. LRG and autophagy inhibitors were used to investigate the protective effect of LRG on pancreatic β-cells *in vivo*. Metabolic indices were measured and pancreatic autophagy was detected. In the INS-1 cells, viability was higher in the FFA + LRG group compared with the FFA group, while the apoptotic rate was lower (P<0.05). The light chain 3B and p62 autophagy-associated proteins were upregulated by LRG, while ATG7 and Beclin1 were downregulated. Autophagy inhibitors reduced the protective effect of LRG in the FFA-treated INS-1 cells. The type 2 diabetes mouse model was successfully established, termed the HF group, in which LRG was observed to reduce body weight and decrease levels of fasting blood glucose, total cholesterol, serum insulin, triglyceride, low density lipoprotein-cholesterol and glycosylated hemoglobin (P<0.05), compared with the HF group. However, chloroquine treatment abrogated these effects (P<0.05, compared with the HF + LRG group; P>0.05, compared with the HF group). Autophagosomes were also observed under ECM in the pancreatic tissues of mice in the HF + LRG group. Therefore, LRG induced autophagy and exerted protective effects on pancreatic β-cells *in vitro* and *in vivo*.

## Introduction

Developing treatment strategies for diabetes, a metabolic disease characterized by a chronic increase in blood glucose levels resulting from defects in insulin secretion, is an acknowledged challenge. As a potential therapeutic drug, glucagon-like peptide 1 (GLP-1) has several physiological benefits, including improving the synthesis and secretion of insulin, inhibiting the secretion of glucagon, promoting the proliferation and genesis of β-cells, inhibiting the apoptosis of β-cells, reducing food intake, slowing gastric emptying, improving insulin utilization in peripheral tissues and reducing the output of glycogen (1). Despite its numerous attributes, natural GLP-1 has a half-life of ~1–2 min and is rapidly degraded by dipeptidyl peptidase IV. As a result of these disadvantages, natural GLP-1 cannot be used directly to treat type 2 diabetes mellitus. Liraglutide (LRG) is a long-acting GLP-1 analogue, which is not degraded as easily as natural GLP-1 and retains the pleiotropic effects of GLP-1, has been reported to prevent multiple risk factors of diabetes by reducing blood glucose, protecting β-cells, reducing body weight, reducing blood pressure and regulating blood lipids (2).

Previous studies have confirmed the safety of LRG and its advantages in treating type 2 diabetes mellitus and preventing cardiovascular diseases (3,4). However, the exact mechanisms involved remain to be elucidated. Palmitic acid may activate an endoplasmic reticulum (ER) stress response in β-cells and induce cellular dysfunction, while autophagy effectively reduces ER stress (5,6). It has been confirmed that autophagy, which caused many physiological accommodations, was closely with pancreatic β-cells in previous studies (7–10). Autophagy may not only protect pancreatic β-cells from apoptosis induced by external stimuli, but it may also maintain the structure, number and functionality of β-cells, and internal homeostasis (11,12).

Autophagy is associated with the autophagy-lysosome pathway, which has been suggested to remove excess or damaged organelles and maintain internal hemostasis, thereby regulating the growth, development and aging of cells, and correcting hypermetabolism (13). Adverse conditions, including oxidative stress and intracellular lipid deposition, may induce autophagy (14). Persistent adverse conditions may even induce metabolic diseases, including type 2 diabetes mellitus, lipid metabolism disorders and metabolic syndrome (14). Previous studies have demonstrated that several factors, including nutrient deficiencies, insulin resistance, energy deficiency, hypoxia, injury, pathogen infection and ER stress, may induce autophagy (15). Autophagy remains at a low level under normal conditions and effective induction of autophagy is critical for stress adaptation (16).

The universally accepted gold standard for the detection of autophagy is the identification of autophagosomes (double-membrane vesicles) and associated subcellular structures. The most commonly used methods in previous studies include detecting the transformation of light chain 3 (LC3) (LC3B/lC3A), a biomarker of autophagy, using western blot analysis, and detecting aggregation of the LC3 expression plasmid, which contains green fluorescent protein (GFP), under a fluorescence microscope. The formation of LC3B and an increase in green fluorescence are objective evidence of the presence of autophagy (17). Knockout of autophagy-associated genes (ATGs) and the use of autophagy inhibitors have been widely used in autophagy inhibition experiments (18). As an autophagy inhibitor with a low toxicity and the ability to inhibit autophagy *in vitro* and *in vivo*, chloroquine has been widely used in investigations associated with autophagy (19).

Another autophagic activity marker protein is nucleoporin p62 (p62). When autophagy occurs, p62 combines with ubiquitinated proteins and binds to LC3B to form a complex, which degrades in autophagolysosomes, resulting in a decrease in the expression of p62 (20). When the activity of autophagy is inhibited, p62 accumulates in the cytoplasm and its expression increases (20). Autophagy protein 5 (ATG5) is another autophagy-associated gene, which is important in the formation and extension of the membrane of autophagosomes (21). The Beclin1 gene, which is a homolog of ATG6, is also a specific gene that is involved in autophagy in mammals. The Beclin1 gene combines with phosphatidylinositol 3-kinase (PI3K) to form a complex, which regulates the activity of autophagy through the regulation of other ATG proteins (22,23). Previous studies have demonstrated that upregulation of Beclin1 may effectively promote autophagy in cells from mammals (24), and reports have indicated that the Beclini1-GFP complex is widely distributed in the cytoplasm throughout the whole body, not restricted to several specific cells (25–28). These results contradict previous observations that Beclin1 is restricted to the trans-Golgi network, suggesting that Beclin1 may have more extensive effects than expected (29). In embryonic experiments, excessive apoptosis was observed in Beclin1-deprived embryos, suggesting that Beclin1 is a potential autophagy inhibitor (30).

ER is inactivated under normal conditions. The three trans-membrane proteins on the ER membrane, inosito1-requiring transmembrane kinase (IRE-1), pancreatic eukaryotic initiation factor-2α kinase-like ER kinase (PERK) and activation transcription factor 6 (ATF6) may bind to GRP78, a molecular chaperone, under normal conditions, exerting no biological activity. PERK, ATF6 and IRE-1 receive ER stress signals and transmit those signals into the nuclear membrane and cytoplasm to assist the adaptation of cells to the initial stress state. In addition, unfolded proteins induce the detachment of GRP78 from the three transmembrane proteins. The interaction of these three transmembrane proteins activates different stress pathways to reduce the accumulation of unfolded and misfolded proteins, to ensure the correct folding of exported proteins (31). The PERK, ATF6 and IRE-1 signaling pathways may activate the survival pathway of ER stress under mild stimulation, while persistent or prolonged ER stress may activate the downstream pro-apoptotic signaling molecule, CHOP/GADD153 (32,33). CHOP/GADD153 is a growth inhibitor which induces DNA damage at a genetic level. Previous studies have demonstrated that increased expression of CHOP significantly reduce the expression of the B-cell lymphoma 2 (Bcl-2) anti-apoptotic protein, reduce the synthesis of glutathione and increase the level of reactive oxygen species in cells, which may in turn induce apoptosis of the cells (34).

Previous studies have demonstrated that GLP-1 activates the cyclic adenosine monophosphate and PI3K pathways to increase the levels of Bcl-2 and Bcl-extra large (Bcl-x1), and reduce the level of caspase-3, thereby inhibiting apoptosis and promoting the proliferation of β-cells (35). It has also been hypothesized that LRG upregulates the production of nitric oxide to improve anti-inflammatory effects, which may protect pancreatic β-cells from apoptosis (36). LRG may also inhibit the expression of adhesion factors and reduce the function of endothelial cells in apolipoprotein E (ApoE)^−/−^ mice (37). Therefore, the protective effects of LRG on pancreatic β-cells have been widely accepted, however, the mechanisms underlying these effects remain to be fully elucidated.

The present study aimed to investigate the protective effects of LRG on pancreatic β-cells. Free fatty acids (FFA) and LRG were administered to pancreatic β-cells, apoptosis was determined and the mechanisms underlying the activation of autophagy by LRG were identified. The effects of autophagy inhibitors on the protective effects of LRG were also investigated. In an ApoE^−/−^ mice diabetic model, the effects of LRG on body weight, blood parameters and the formation of autophagosomes were measured to evaluate the level of autophagy.

## Materials and methods

### Cell line

The INS-1 insulinoma pancreatic islet cell line was purchased from the Type Culture Collection of the Chinese Academy of Sciences (Shanghai, China) and was cultured at 37°C with 5% CO_2_. The cells (1×10^6^) were seeded into 6-well plates and treated with either 1 mM FFA (Gibco Life Technologies, Carlsbad, CA, USA), 80 nM LRG (Novo Nordisk Company, Aalborg Øst, Denmark) or 50 *µ*M chloroquine Sigma-Aldrich, St. Louis, MO, USA).

### MTT assay

At a confluence of 75–80%, 0.25% trypsin was used to digest the cells (37°C for 1 min). RPMI-1640 culture medium (Gibco Life Technologies, Carlsbad, CA, USA), containing 10% fetal bovine serum was added when the cells began to shrink and revert. The cells were resuspended and the density was adjusted to 5×10^4^/ml. The cells were cultured in a 96-well culture plate (100 *µ*l/well) overnight at 37°C to allow attachment. The cells were then treated with either normal culture medium (CON group), FFA (FFA group), LRG (LRG group) or LRG and FFA (FFA + LRG group) for 36 h. Each group contained three or four repeats. Following treatment, MTT (20 *µ*l/well) was added, the culture medium was carefully removed, and 150 *µ*l dimethyl sulfoxide was added to each well. The culture plate was then placed on a shaker and agitated at a low speed (18 × g) for 10 min. The absorbance density (490 nm) of each well was measured using an enzyme-linked immunometric meter (Modulus™; YPH BIO technology, Ltd., Beijing, China) at 490 nm.

### Annexin V/propidium iodide (PI) double staining flow cytometry

The cells (2×10^5^) were stained with PI and fluorescein isothiocyanate-annexin V (Sigma-Aldrich) for 15 min and the percentages of apoptotic cells were analyzed by flow cytometry using a BD FACSVerse™ flow cytometer (BD Biosciences, Franklin Lakes, NJ, USA).

### ECM

The cells in each group were treated for 36 h, as described above, following which 0.25% trypsin was used for collection. The cells were placed in an Eppendorf tube for fixation (fixative provided by Weiya Electron Microscopy Center, Shanghai, China), then were centrifuged at 5,000 × g for 15 min and transferred to the Weiya Electron Microscopy Center for observation, imaging and analysis.

### Plasmid transfection

The LC3 expression plasmid containing GFP, provided by Professor Jin Shengkan at the University of Medicine and Dentistry of New Jersey (New Brunswick, NJ, USA) was transfected into the INS-1 cells in the different treatment groups using Lipofectamine™ 2000 Transfection reagent (Gibco Life Technologies). A fluorescence microscope (CX41-32RFL; Olympus Corporation, Tokyo, Japan) was used to detect the localization of GFP in the cultured cells and determine the fluorescence, reflecting autophagy.

### Immunoblotting

Whole cell lysates were prepared in radio-immunoprecipitation assay buffer (Beyotime Institute of Biotechnology, Haimen, China). The protein content was determined using a Bio-Rad Protein Assay kit (Bio-Rad Laboratories, Inc., Hercules, CA, USA) with 5% bovine serum albumin (Gibco Life Technologies). Equal quantities of protein were separated using 8% SDS-PAGE (Shanghai Reagent Manufactory, Shanghai, China) and electro-transferred onto polyvinylidene fluoride membranes. Following blocking with 5% non-fat milk, the membranes were incubated with the following primary antibodies: anti-LC3B antibody (2775S; Cell Signaling Technology, Inc., Danvers, TX, USA), anti-CHOP antibody YT0912; ImmunoWay Biotechnology Company, Newark, DE, USA), anti-GRP78 (G9043), anti-p62 (N1163), anti-ATG7 (A2856) and anti-Beclin1 antibodies (SAB5300513) (Sigma-Aldrich) at 25°C for 2 h. The membranes were then incubated with the following secondary antibody: Goat anti-mouse immunoglobulin G (01-18-02; Shanghai Reagent Manufactory, Shanghai, China), at 25°C for 2 h. Subsequent to washing with Tris-buffered saline with 0.1% Tween-20 (Shanghai Reagent Manufactory), signals were detected using an Enhanced Chemiluminescence kit (GE Healthcare Bio-Sciences, Pittsburgh, PA, USA).

### In vivo experiments

Healthy 6-week-old specific pathogen-free male ApoE^−/−^ mice (n=36; body weight, 19.12±1.42 g) were purchased from the Animal Center of Peking University Health Science Center [Beijing, China; license number, SCXK (jing) 2011–2012]. The mice were maintained at 25°C. Animal care was provided in accordance with the Guidelines for the Care and Use of Laboratory Animals (38), and all experiments were approved by the Ethics Committee of the Jinan Military Area General Hospital of People's Liberation Army. The baseline body weights of the mice were similar among the groups and the animals were allowed free access to food and water. The mice were randomly divided into four groups, each containing nine mice. The CON group received normal maintenance food (mass proportion: 100% basic food; energy proportion: 10% fat, 19% protein and 71% carbohydrate), while the HF group received high-fat food (mass proportion: 68% basic food, 20% lard, 2% cholesterol, 10% yolk powder and 0.1% cholate; energy proportion: 56% fat, 9% protein and 34% carbohydrate). The HF + LRG group received high-fat food and LRG; and the HF + LRG + CQ group received high-fat food, LRG and chloroquine. With the exception of mice in the CON group, all animals received a 50 mg/kg STZ injection subsequent to being fed a diet of high-fat food for 8 weeks, and blood was collected using a tail-cut method 3 days later. A randomly detected fasting blood glucose (FBG) ≥11.1 mmol/l was considered a successful model. The mice in the CON and HF groups received an intraperitoneal injection of normal saline (5 *µ*l/g, twice/day) for 30 days, mice in the HF + LRG group received intraperitoneal injections of LRG (1 mg/kg, twice/day) for 30 days, and mice in the HF + LRG + CQ group received intraperitoneal injections of LRG (1 mg/kg, twice/day) and chloroquine (50 mg/ml, once/3 days) for 30 days. The body weight, food intake and water intake of each mouse was measured every 2 weeks. All mice were sacrificed 30 days following LRG/CQ administration, prior to the collection of blood samples.

### Collection of biological parameters

FBG was determined using the glucose oxidase method, fasting insulin (FINS) and glycated hemoglobin (GHb) were determined using enzyme-linked immunosorbent assay, plasma triglyceride (TG), total cholesterol (TC), low density lipoprotein cholesterin (LDL-C), high density lipoprotein cholesterin (HDL-C) and FFA levels were determined using enzymatic methods.

### Statistical analysis

SPSS software, version 16.0 (SPSS Inc., Chicago, IL, USA) was used for statistical analyses. All data are expressed as the mean ± standard deviation. Analysis of variance was used for comparison among three or more different groups, Student's t-test was used for comparison between two groups and non-parametric analysis was used for data with unequal variances. P<0.05 was considered to indicate a statistically significant difference.

## Results

### LRG reduces the apoptosis of INS-1 cells induced by FFA

**Figure 1:**
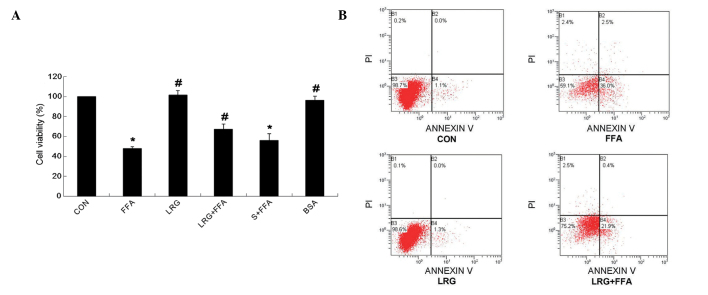
LRG reduces apoptosis of INS-1 cells induced by FFA. (A) Viabilities of INS-1 cells treated with normal saline (CON group), LRG or FFA + LRG for 24 h. MTT assays were performed and the cell viability relative to that of the CON group was measured. All data are expressed as the mean ± standard deviation. (B) Apoptotic rates in the CON, FFA, LRG and FFA + LRG groups were detected using annexin V/PI double labeling flow cytometry. CON, control; FFA, free fatty acids; LRG, liraglutide; PI, propidium iodide; S, saline; BSA, bovine serum albumin.

As shown in Fig. 1, following treatment of the INS-1 cells with normal culture medium (CON group), FFA (FFA group), LRG (LRG group) or LRG and FFA (FFA + LRG group), the cell survival rate was significantly higher in the FFA + LRG group, compared witht he FFA group (P<0.05; Fig. 1A). The annexin V/PI double labeling flow cytometric analysis demonstrated that the apoptotic rate was significantly lower in the FFA + LRG group, compared with the FFA group (P<0.05). These observations suggested that LRG treatment reduced the INS-1 cell apoptosis induced by FFA.

### LRG protects INS-1 cells by activating autophagy

**Figure 2:**
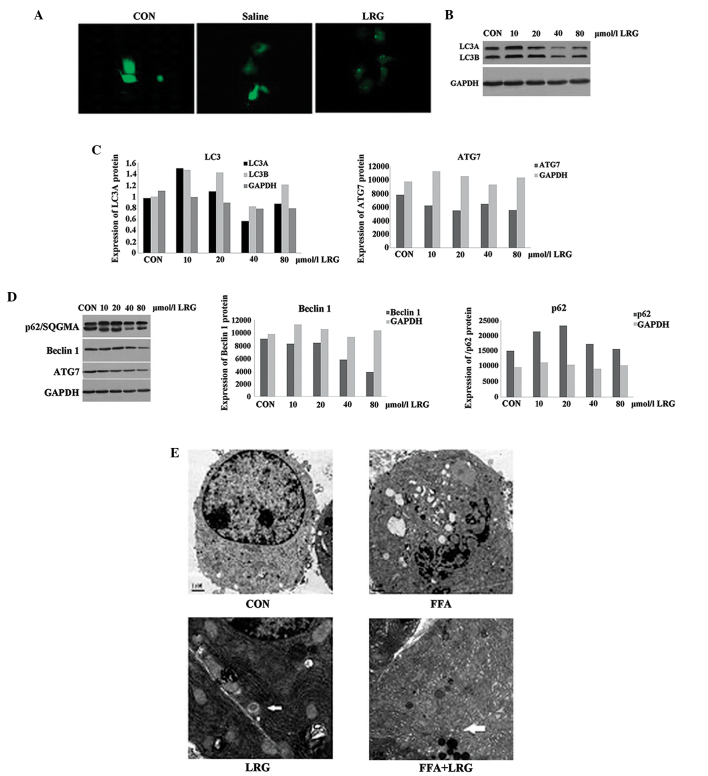
LRG protects INS-1 cells by activating autophagy. (A) INS-1 cells were transfected with the GFP-LC3 expression plasmid and then treated with normal saline or LRG. Cells were observed under fluorescence microscopy (magnification, ×200). (B) INS-1 cells were transfected with the GFP-LC3 expression plasmid and then treated with different concentrations of LRG: L1, 10; L2, 20; L3, 40 or or L4, 80 *µ*mol/l for 36 h. The LC3 protein was detected using immunoblotting. (C) Gray scanning of LC3A and LC3B normalized to that of GAPDH. (D) INS-1 cells were transfected with the GFP-LC3 expression plasmid and were then treated with different concentrations of LRG for 36 h. p62, The ATG7 and Beclin1 proteins were detected using immunoblotting and gray scanning, normalized to that of GAPDH; (E) ECM images of INS-1 cells in the CON, FFA, LRG and FFA + LRG groups. Arrows indicate autophagosomes (magnification, ×8,000). LRG, liraglutide; GFP, green fluorescent protein; LC3, light chain 3; CON, control; FFA, free fatty acids.

Compared with the cells in the CON group, higher levels of green fluorescence were observed in the INS-1 cells treated with LRG (Fig. 2A). LC3B, an indicator of autophagy, was increased in INS-1 cells treated with 20 and 80 *µ*mol/l LRG for 36 h (Fig. 2B and C). No changes were observed in the expression of p62, another autophagy indicator, in the LRG-treated INS-1 cells, however, the expression levels of ATG7 and Beclin1 were significantly reduced (Fig. 2D). The ECM results demonstrated the presence of LRG-induced autophagosomes in the ISN-1 cells. Multiple vesicular bodies with double membranes were present in the LRG and FFA + LRG groups (arrows in Fig. 2E). Excessive and aged organelles were identified inside the bodies; and ER swelling and proliferation were also observed. No autophagosomes were observed in the CON or FFA groups (Fig. 2E). Taken together, these observations suggested that treating INS-1 cells with LRG significantly activated autophagy in the cells.

### CQ AND 3-MA autophagy inhibitors significantly reduce the protective effects of LRG

**Figure 3:**
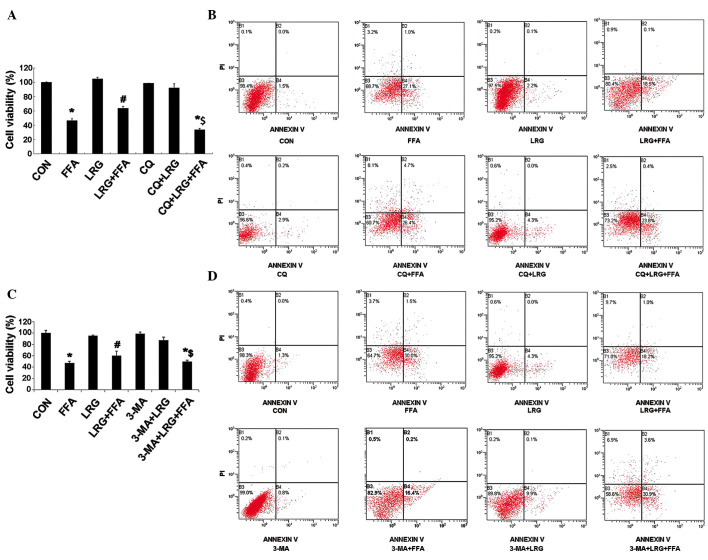
CQ and 3-MA autophagy inhibitors significantly reduce the protective effects of LRG. (A) Viabilities and (B) apoptotic rates of the INS-1 cells treated with normal saline (CON group), FFA, LRG, FFA + LRG, CQ, CQ + LRG or CQ + FFA + LRG for 24 h. (C) Viabilities and (D) apoptotic rates of the INS-1 cells treated with normal saline (CON group), FFA, LRG, FFA + LRG, 3-MA, 3-MA + LRG and 3-MA + FFA + LRG for 24 h. CQ, chloroquine; 3-MA, 3-methyladenine; LRG, liraglutide; CON, control; FFA, free fatty acids; PI, propidium iodide.

The viability of the INS-1 cells in the CON, FFA, LRG, FFA + LRG, CQ, CQ + FFA, CQ + LRG and CQ + FFA + LRG groups were determined using MTT assays, and the results demonstrated that the cell viability in the CQ + FFA + LRG group was significantly lower than in the FFA + LRG group (P<0.05; Fig. 3A). Annexin V/PI double labeling flow cytometry indicated that the apoptotic rate was significantly higher in the CQ + FFA + LRG group than in the FFA + LRG group (P<0.05; Fig. 3B). Another autophagy inhibitor, 3-MA, was also used, instead of CQ, in the present study to confirm these observations, and the results indicated that the cell viability in the 3-MA + FFA + LRG group was significantly lower than in the FFA + LRG group (P<0.05; Fig. 3C). The results of the annexin V/PI double labeling flow cytometry produced similar results in the 3-MA and CQ groups (Fig. 3D). These observations demonstrated that autophagy inhibitors were able to effectively reduce the protective effects of LRG on the apoptosis of INS-1 cells induced by FFA.

### ER stress is involved in activating autophagy of INS-1 cells, induced by LRG

**Figure 4:**
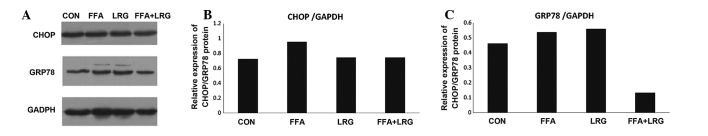
Endoplasmic reticular stress is involved in the activation of autophagy of INS-1 cells induced by LRG. INS-1 cells were treated with normal saline (CON), FFA, LRG and FFA + LRG for 24 h. (A) CHOP and GRP78 proteins were detected using immunoblotting, the results were normalized to that of GAPDH. Quantification of the expression levels of (B) CHOP and (C) GRP78. LRG, liraglutide; CON, control; FFA, free fatty acids.

As shown in Fig. 4, the levels of GRP78 and CHOP, two ER stress-associated proteins, were determined using immunoblotting. The results revealed that the expression levels of GRP78 and CHOP were increased significantly in the INS-1 cells treated with FFA, while treatment with LRG significantly reduced the expression of GRP78 and CHOP, compared with CON treatment. These observations suggested that LRG protected pancreatic β-cells from FFA-induced apoptosis by activating autophagy to overcome ER stress.

### LRG reduces body weight and improves glucolipid metabolism, which is reversed by the inhibition of autophagy

**Figure 5:**
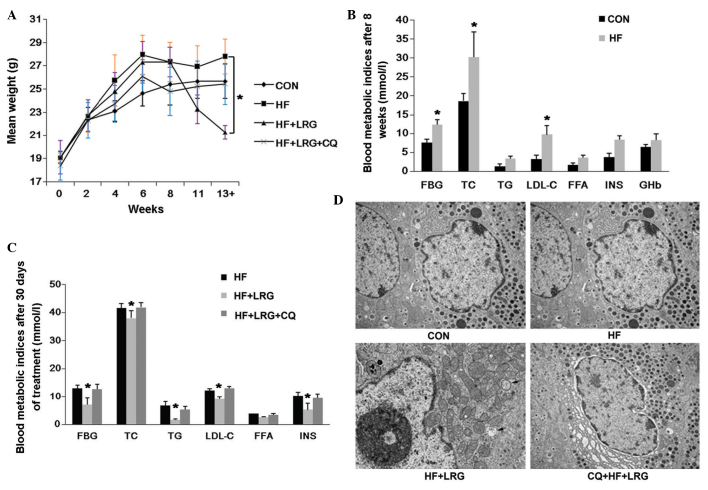
LRG reduces the body weight and improves glucolipid metabolism of the mice, and these effects are reversed by the inhibition of autophagy. (A) Body weights of mice in the normal diet fed group (CON), mice fed with a high-fat diet for 8 weeks and injected with streptozotocin (HF), mice fed a high-fat and treated with intraperitoneal injection of LRG (1 mg/kg, twice/day) for 30 days (HF + LRG) and mice fed with a high-fat and treated with intraperitoneal injection of LRG (1 mg/kg, twice/day) and CQ (50 mg/ml, once/3 days) for 30 days (HF + LRG + CQ). (B) Blood metabolic indices of mice in the CON and HF groups after 8 weeks of a normal or high-fat diet. (C) Blood metabolic indices of mice in HF, HF + LRG and CQ + HF + LRG groups after 30 days treatment with either normal saline, LRG or LRG + CQ. (D) Electron microscopy images of pancreatic tissues of mice in the CON, HF, HF + LRG and CQ + HF + LRG groups. Arrows indicates autophagosomes (magnification, ×8,000). All data are expressed as the mean ± standard deviation. LRG, liraglutide; CON, control; HF, high-fat; CQ, chloroquine; FBG, fasting blood glucose; TC, total cholesterol; TG, plasma triglyceride; LDL-C, low density lipoprotein cholesterol; FFA, free fatty acids; INS, fasting insulin; GHb, glycated hemoglobin.

The results of the *in vivo* experiments are shown in Fig. 5. The body weights of the mice in the HF + LRG group were significantly lower than in the HF group (P<0.05); however, the difference between the HF + LRG + CQ group and HF group was not statistically different (P>0.05; Fig. 5A). Following an 8-week high-fat diet and intraperitoneal injection of STZ, the levels of FBG, TC and LDL-C were significantly higher in the ApoE^−/−^ mice in the HF group, compared with those in the CON group (P<0.05); no significant differences were observed in body weight, TG, FFA, INS and GHb between these two groups (P>0.05; Fig. 5B). Following treatment of the mice in the different groups for 30 days, the levels of FBG, TC, INS, TG, LDL-C and GHb were significantly lower in the HF + LRG group, compared with those in the HF group (P<0.05), however, no significant difference was observed in the level of FFA. No significant differences in the parameters were observed between the HF + LRG + CQ group and HF group (P>0.05).

ECM examinations were also performed, and one image was selected from each of the CON, HF, HF + LRG and HF + LRG + CQ groups. No autophagosome-like micro-structures were identified in the images of the CON group, however, several vesicular bodies with double membranes were observed in the HF + LRG group, and excessive and aged organelles were identified inside these bodies. These observations demonstrated that LRG treatment induced the formation of autophagosome in the pancreas of the ApoE^−/−^ mice. The results also indicated that, in mice in the HF + LRG + CQ group, autophagy was inhibited and no autophagosomes were identified. These findings suggested that LRG activated autophagy in a high-fat high-glucose environment and inhibited the apoptosis of the pancreatic β-cells, whereas the inhibition of autophagy decreased the protective effects of LRG on the cells.

## Discussion

Treating diabetes with GLP-1 has received increased attention, and the long-acting GLP-1 analogue, LRG, exhibits the pleiotropic effects of GLP-1, but is not as easily degraded (1). Autophagy may not only protect pancreatic cells from apoptosis induced by external stimuli, but also maintains the number, structure and functionality of pancreatic β-cells, and maintains internal homeostasis (11,12,39). ApoE^−/−^ mice have a high risk of developing lipid metabolism disorders and have been reported to rapidly develop hyperlipidemia and atherosclerosis. Previous studies have demonstrated that estrogen has protective effects on lipid metabolism, therefore, to evaluate the protective effects of LRG on pancreatic β-cells, the present study used male ApoE^−/−^ mice, which were fed a high-fat diet to induce a high lipid model, and STZ injections were administered to the mice to induce a mouse model of diabetes (40).

The mechanisms by which LRG preserves pancreatic β-cells have been investigated in other murine diabetes models. In a previous study to determine the molecular mechanism by which LRG preserves pancreatic β-cell mass, obese diabetic db/db mice were treated with LRG for either 2 days or 2 weeks, while mice with normal glycemic levels were treated with LRG for 2 weeks. LRG was found to modify the expression levels of genes associated with cell apoptosis, including Bcl2, caspase 8, caspase 3 and cadherin in db/db mice. However, the mRNA levels of these genes were not altered in mice with normal glycemic levels during short- or long-term treatment. These observations suggested that LRG directly suppressed β-cell apoptosis in mice under hyperglycemic conditions (41). Another study evaluated the protective effects of LRG in C57B1/6 J mice, in which the mice were provided with drinking water treated with either corticosterone or a vehicle for 5 weeks. The mice were then administered with once-daily injections of either PBS or LRG and, in the C57B1/6 J diabetes model, LRG promoted the function of the β-cells and slowed the development of hyperglycemia (42).

The results of the cellular experiments in the present study demonstrated that LRG protected the pancreatic β-cells from FFA-induced apoptosis by activating autophagy, which is associated with ER stress in cells. CQ and 3-MA, two autophagy inhibitors, significantly reduced the protective effects of LRG. The results of the animal experiments in the present study also demonstrated that LRG reduced the body weight of the mice and improved the glucolipid metabolism, following feeding of the mice with a high-fat diet. However, the protective effects disappeared when autophagy was inhibited. LRG also improved blood glucose and regulated blood lipids in the diabetes model. These improvements were significantly reduced following the inhibition of autophagy, suggesting LRG protected the pancreatic β-cells by activating autophagy.

In the present study, the expression of GRP78, an ER stress switch protein, was increased in the FFA group, compared with the CON group, which activated the ER stress pathway and increased the expression of CHOP, in turn activating the downstream pathway of apoptosis. The expression of GRP78 was significantly reduced following the administration of LRG, and no excessive ER stress was observed. The levels of CHOP and cellular apoptosis were also significantly reduced. These observations were in accordance with previous findings, which indicated that LRG protects pancreatic β-cells from apoptosis by activating autophagy (43). However, the expression levels of GRP78 and CHOP were not significantly different between the CON and LRG groups, suggesting LRG did not activate the ER stress pathway and inhibit the expression of apoptotic factors, unless the apoptosis pathway had not already been activated. Previous studies have also demonstrated that ER stress is involved and assists in protecting the body against the adverse effects induced by damage; however, in certain cases the damage was too severe for ER homeostasis to be maintained, and subsequent apoptotic signal activation results in ER-associated death (44–46). A previous study demonstrated that ER stress also mediates another pathway of cell death, namely autophagy (6), which is in accordance with the observations of the present study.

ApoE^−/−^ mice received a high-fat diet for 8 weeks followed by intraperitoneal injection of STZ to induce a diabetes model, and LRG was administered for 30 days. The body weight, FBG, TC, INS, TG and LDL-C of the mice in the HF + LRG group were significantly lower, compared with those in the HF group (P<0.05), while no significant difference was observed in FFA between these two groups. High concentrations of FFA induced insulin resistance and impaired the insulin producing function of pancreatic β-cells. In the present study, LRG was unable to effectively reduce the levels of FFA, suggesting that, although LRG improved blood glucose, regulated blood lipids and reduce body weight, it did not improve the sensitivity to insulin, which is a limitation of LRG.

In conclusion, the *in vitro* and *in vivo* experiments performed in the present study provided results, which improve the current understanding of the mechanisms involved in the protective effects of LRG on pancreatic β-cells, and provided evidence for the prevention of β-cell apoptosis in clinical practice.
